# Detecting Human Presence at the Border of the Northeastern Italian Pre-Alps. ^14^C Dating at Rio Secco Cave as Expression of the First Gravettian and the Late Mousterian in the Northern Adriatic Region

**DOI:** 10.1371/journal.pone.0095376

**Published:** 2014-04-23

**Authors:** Sahra Talamo, Marco Peresani, Matteo Romandini, Rossella Duches, Camille Jéquier, Nicola Nannini, Andreas Pastoors, Andrea Picin, Manuel Vaquero, Gerd-Christian Weniger, Jean-Jacques Hublin

**Affiliations:** 1 Department of Human Evolution, Max Planck Institute for Evolutionary Anthropology, Leipzig, Germany; 2 Universitá di Ferrara, Dipartimento di Studi Umanistici, Ferrara, Italy; 3 Neanderthal Museum, Mettmann, Germany; 4 Institut Català de Paleoecologia Humana i Evolució Social (IPHES), Tarragona, Spain; 5 Museo delle Scienze, Trento, Italy; 6 Universitat Rovira I Virgili, Area de Prehistòria, Tarragona, Spain; University of Florence, Italy

## Abstract

In the northern Adriatic regions, which include the Venetian region and the Dalmatian coast, late Neanderthal settlements are recorded in few sites and even more ephemeral are remains of the Mid-Upper Palaeolithic occupations. A contribution to reconstruct the human presence during this time range has been produced from a recently investigated cave, Rio Secco, located in the northern Adriatic region at the foot of the Carnic Pre-Alps. Chronometric data make Rio Secco a key site in the context of recording occupation by late Neanderthals and regarding the diffusion of the Mid-Upper Palaeolithic culture in a particular district at the border of the alpine region. As for the Gravettian, its diffusion in Italy is a subject of on-going research and the aim of this paper is to provide new information on the timing of this process in Italy. In the southern end of the Peninsula the first occupation dates to around 28,000 ^14^C BP, whereas our results on Gravettian layer range from 29,390 to 28,995 ^14^C years BP. At the present state of knowledge, the emergence of the Gravettian in eastern Italy is contemporaneous with several sites in Central Europe and the chronological dates support the hypothesis that the Swabian Gravettian probably dispersed from eastern Austria.

## Introduction

Numerous sites throughout the Italian Peninsula and the western Balkans document key events between the late Middle Palaeolithic and the Mid-Upper Palaeolithic. Focusing on the northern Adriatic Sea rim which includes the Venetian region and the Dalmatian coast, the millennia preceding the demise of Neanderthals are recorded in very few sites which displayed data of variable relevance [1]–[3]. Settlements were logistically structured in accordance with the vertical displacement of economic activities at mountain districts sheltered sites were repeatedly used to accomplish different types of complex tasks or were inhabited for short-term occupations, as it has been suggested from the fractionation of stone tool production sequences [3]. Flint provisioning and lithic economy was therefore fully organized and reveal how human land-use varied accordingly to the geographical location and function of the sites [3].

Even scarcer in this area is the archaeological evidence of the Mid-Upper Palaeolithic, a period better known along the Tyrrhenian Sea and the southern Adriatic coasts, where evidence of intense Gravettian occupation can be found [4].

One of the most debated issue is whether the Gravettian developed from a local Aurignacian [5]–[8] or results from immigration or cultural diffusion processes through various corridors between European regions [4], [9]–[11]. This paper will not enter into this broader issue, instead it will deal with the Northern Italian evidence and the role of two possible passageways, one from the west (France) and one from the east (Balkan region) [9], [12]–[14].

**Figure 1:**
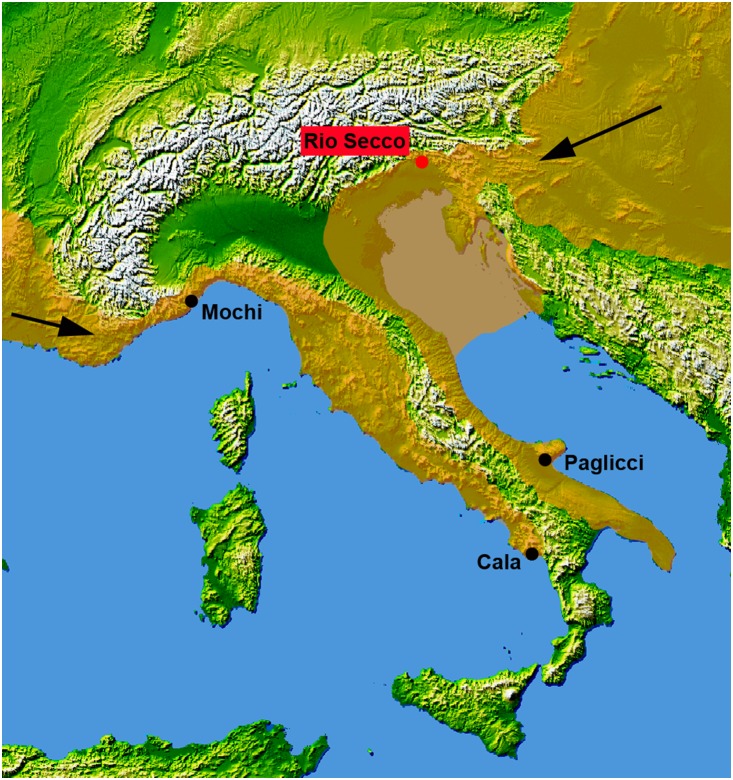
The earliest Mid-Upper Palaeolithic sites in Italy. Rio Secco is marked in red, the sea level is at −80 m (Base map from NASA http://www2.jpl.nasa.gov/srtm/world.htm) [20].

The earliest Italian Gravettian groups is documented around 28,000 ^14^C BP in Paglicci Cave in the southern end of the Peninsula [15]–[17], and the majority of the sites, adjacent to the two opposite Italian coasts, are recorded at 26,000–24,000 ^14^C BP (Figure 1) [12].

All along the Tyrrhenian coast, the lithic assemblages described at Mochi rockshelter and La Cala Cave suggest an influence from the French Gravettian [9], [12], [18]. In contrast, the conspicuous Gravettian evidence from Paglicci Cave, in the South Adriatic area, shows discrepancy with the Tyrrhenian belt from a technological point of view. This indication suggest signatures of cultural influence from a possible eastern route starting from the Carpathian basin [9] and crossing the trans-Adriatic region, when the sea level at that time was estimated at about 80 m lower than present day [19], [20] (Figure 1). Nevertheless, evidence across the Adriatic coast is still too scanty, mostly due to ephemeral field researches, aside a reduced number of sites; e.g. Broion Rockshelter and Fonte delle Mattinate and the above mentioned Paglicci Cave [11], [21]–[23].

Moreover, Paglicci is not the key site to understand the issue of the local development of the Italian Gravettian because Aurignacian and Early Gravettian assemblages show an abrupt change with neither transitional nor formative characters [11], [14].

As it is shown the Gravettian settlement of Italy is spatially sparse; in this context the recently investigated cave of Rio Secco, located in the northern Adriatic region, provides evidence on the late Mousterian and the earliest Gravettian, due to a set of new radiometric dates on bone and charcoal samples. Considering its geographic setting between the upper Adriatic Plain and the Pre-Alps, Rio Secco Cave holds a strategic position to investigate the mobility pattern of the Palaeolithic hunter-gatherers across the natural corridor between the Italian Peninsula and the Carpathian Basin.

## Rio Secco Cave Consideration

### The Site of Rio Secco Cave

**Figure 2:**
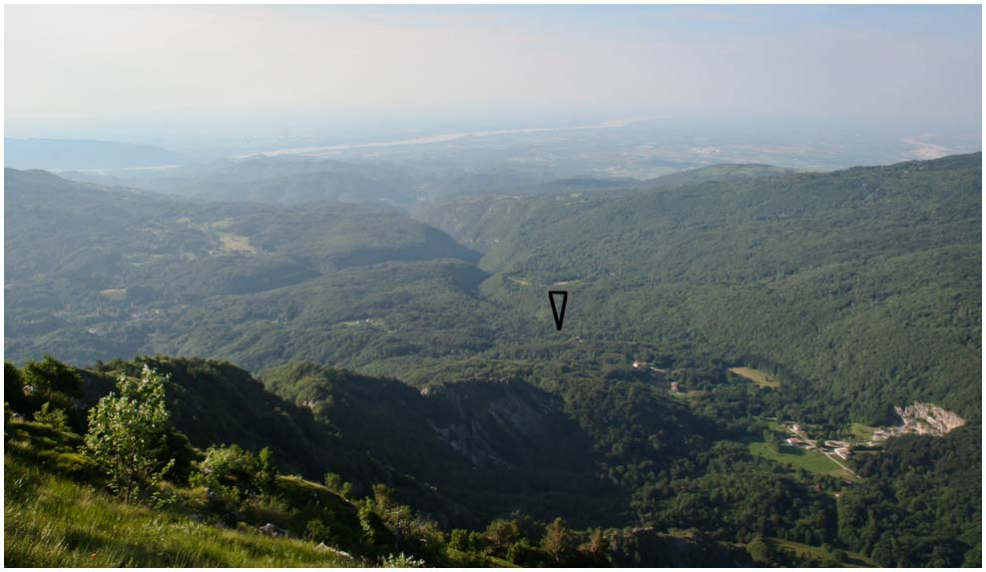
Southern view on the Pradis plateau from the Mount Rossa edge (1,309 m). The position of Rio Secco Cave in the gorge is marked. Background, the alluvial plain crossed from the Tagliamento River at the center.

Rio Secco Cave is situated in the northeastern portion of the Italian Peninsula, near the village of Clauzetto (Pordenone), at 580 m asl on the Pradis Plateau in the eastern part of the Carnic Pre-Alps. The Pradis Plateau comprise an area of 6 sq km, enclosed on three sides by mountains peaking from 1,148 m to 1,369 m and to the south by the foothills, facing the present-day Friulian Plain (Figure 2). Rio Secco Cave is a large sheltered cave opening on the left slope of a stream gorge at about 20 m above the present day stream bed. Facing south, the shelter has a wide and flat roof derived from the collapse of large slabs of the stratified limestone. The sheltered area is enclosed from the outside by a ridge of large boulders. The cave opens in the middle of the wall and continues as a gallery for 12 m until the sediments completely fill it up. In the outer area the fill forms a slope-waste deposit thickening along the present day drip line where the boulders define the original extension of a vast roof.

The presence of Palaeolithic settlements at Rio Secco Cave was detected in 2002 after a test-pit [24] and an archaeological excavation has been carried out at the site since 2010.

### Stratigraphy

**Figure 3:**
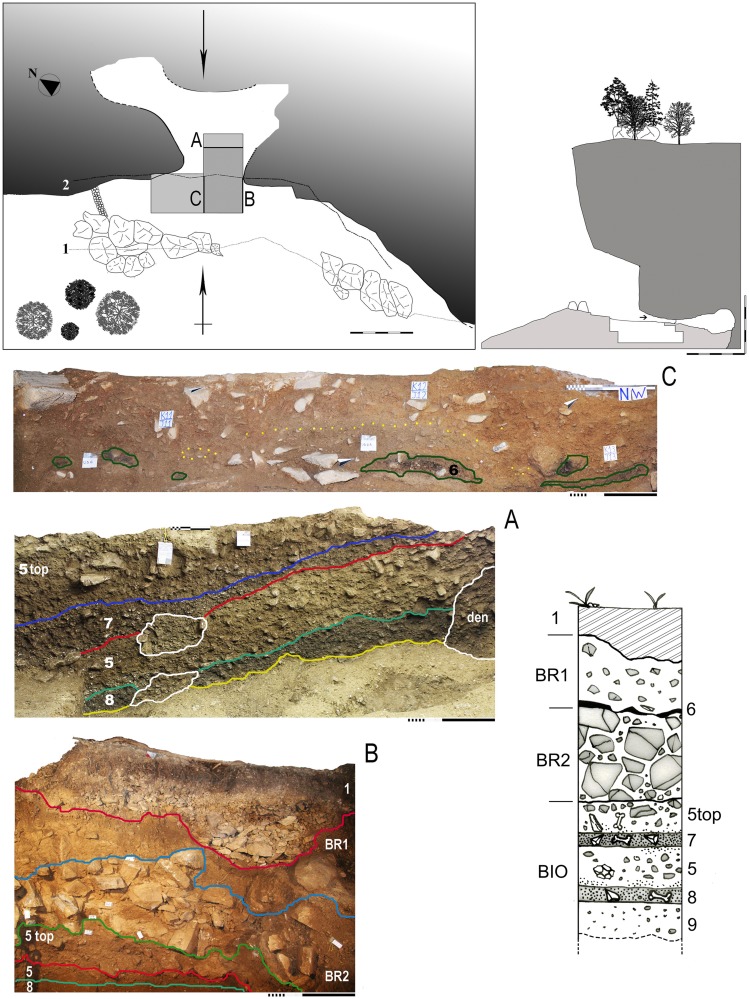
Sketch map and section of the site. Position of the excavated area and the stratigraphic exposures: A – section showing portions of layer 6 embedded in macro-unit BR1; B - section showing the Mousterian layers from 5 top to 8; C – the main sagittal section exposed in 2010 with the reworked sediment sealing the Mousterian sequence from 5 top to 8 (after [25]).

The cave is filled with an ensemble of sedimentary bodies of differing volume, shape, composition and origin, grouped into four macro-stratigraphic units and separated by erosional and sedimentary discontinuities [25]. From the top, the macro-units are 1, which originated during historical times, BR1, BR2 and BIO1 (Figure 3).

Macro-unit BR1 includes layer 4 and an anthropic horizon containing Gravettian flint artifacts, layer 6. The most relevant features are angular to subangular stones, with fragments of karst limestone pavement that originated from the collapse of the vault. Layer 6, with organic matter and micro-charcoal has been exposed at the entrance of the cave shelter, approximately 20 cm below the top of BR2: it is thin, planar, discontinuous, and contains rare bones and lithics (Figure 3).

Macro-unit BR2 is a massive open-work stone-supported breccia made of angular boulders and randomly deposited stones. It lies in the external zone but ends 1 m behind the drip line in the SE zone of the cavity, where it seals the layer 5 top. Large patches have been reworked by marmots, as demonstrated by bones, an articulated skeleton found within the tunnels, several burrows and dens.

The sedimentary body below BR2 is composed of stones and loamy fine fraction and is labeled BIO1 due to the intense bioturbation caused by the activity of marmots, responsible for mixing, displacing portions of anthropic sediment, and scattering Mousterian flint implements, bones and charcoals. At the top of this macro-unit, one finds layer 5 top, a brown level of variable thickness with archaeological content. Due to its variable thickness, layer 5 top has been locally divided in two arbitrary cuts, I and II. Below, the loamy, dark yellowish-brown layer 7 has been found only in some squares under the cave vault and not in the external zone, where it is cut by the burrows. The upper boundary with layer 5 top is marked by an increasing frequency of bones and lithics, some of which also bear signatures of accidental heating. Sandwiched between the two anthropic horizons layers 7 and 8, layer 5 is made of stones and loamy fine fraction with dispersed bones and lithic implements frequently affected from post-depositional alteration. Layer 8 continues in the inner cavity and is best described as 10 cm thick, stony, with dark brown loamy fine fraction, frequent tiny charcoals, small and burnt bones. Layer 8 lies over layer 9, possibly a fifth macro-unit, made of stones and yellowish brown sandy-loam, with no charcoal or other finds.

### Cultural Sequence

**Figure 4:**
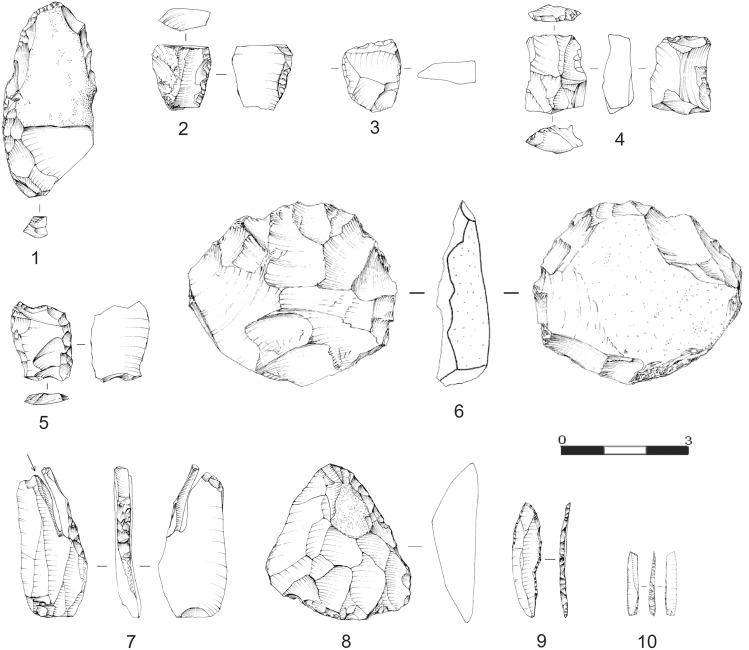
Lithic implements from Mousterian layers 8, 7, 5 and 5 top. Mousterian layers 8 (1), 5 (6), 7 (2, 3) and 5 top (4, 5). Scraper (1), scraper shortened by distal truncation and thinned on the dorsal face (2), core-edge removal flake from discoid core (3), bladelet core (4), double scraper shortened by proximal truncation (5), Levallois centripetal core (6). Gravettian implements: burin with refitted burin spall (7), end-scraper on large retouched flake (8), possibly unfinished backed point (9), double truncated backed bladelet (10). Drawn by S. Muratori.

The archaeological contents of BR1 and BIO1 include numerous lithic artifacts ascribed to the Middle Palaeolithic (layers 5 top, 7, 5, 8) and Upper Palaeolithic (layer 6 and correlated arbitrary cuts 4c and 4d) and a few bone retouchers [25]. The Mousterian assemblages are characterized by the use of Levallois and discoid technologies (Figure 4). Layer 8 has yielded scrapers of variable type and size and flakes with patterns typical of Levallois technology. Layer 5 has produced evidence of the use of Levallois technology as well, represented by recurrent unipolar flakes and centripetal flakes and cores, of discoid technology represented from core-edge removal flakes and pseudo-Levallois points and retouched tools, mostly scrapers. Layer 7 has produced flakes and a few tiny scrapers. In layer 5 top lithic items are varied: Levallois and discoid flakes, short blades and short bladelet cores. The Upper Palaeolithic of layer 6 consists of a handful of pieces technologically characterized by blade/bladelet production. The tools are three burins on truncation made on blades and on rejuvenation blades (Figure 4). One of them shows remarkable negatives of several burin spalls, of which one was refitted and for this reason it should be interpreted as a bladelet core. In addition, there are two end scrapers produced on cortical flakes, one of which is thick and large. Among the projectile pieces, we count one backed bi-truncated bladelet, one possibly unfinished backed point and one undeterminable backed fragment.

**Figure 5:**
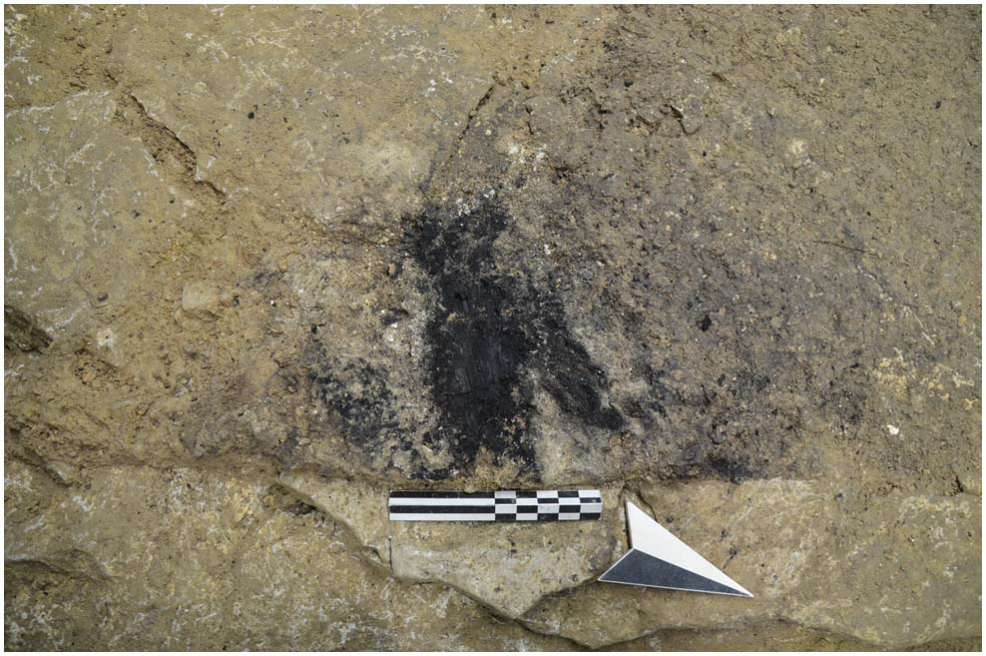
US6_SI hearth brought to light at the entrance of the cave.

Evidence for the use of fire has been found in layers 8 and 7 by tiny dispersed charcoals, burnt bones and heat-affected flints. In layer 6 two hearths have been brought to light, even if partially affected from post-depositional disturbances, labeled as US6_SI and US6_SII. The former is an agglomeration of charcoals mostly disaggregated around a large piece of charred wood (Figure 5). This hearth has been cut by illegal excavations in the back of the cave. Traces of ash are lacking, but there is a thin reddish horizon below the level of charcoals. The hearth US6_SII is a small agglomeration of charcoal largely disturbed by several interlaced burrows.

### Faunal Remains

Every stratigraphic unit contained animal bone remains. The colonization of the cave fill by marmots is clearly documented by diagnostic signatures observed in BR1 and BR2, such as dens, chambers and articulated skeletons. There are fewer faunal remains in the Gravettian layers in comparison with the Mousterian.

The archaeozoological analysis, still in progress, reveals among the ungulates the presence of caprids (*Capra ibex* and *Rupicapra rupicapra*) and remains of *Bos*/*Bison* (*Bison priscus/Bos primigenius*). Traces of human modification on the bones include cut-marks on shafts of caprids, partly combusted, and on a marmot clavicle. One partially burned epiphysis of the scapula of *Castor fiber* has been found associated to the hearth US6_SI.

In the Mousterian sequence, carnivores (brown bears, cave bears, mustelids and canids) predominate over the ungulates, which rather than caprids (chamois and ibex) or bovids, consist more of cervids such as red deer, roe deer, elk and wild boar (Peresani et al., in press). Bones are mostly fragmented, due to post-depositional processes as well as human and carnivore activity. Human interest in ungulates is evidenced by cut marks on red deer. Also the remains of Ursus spelaeus and Ursus sp. from layers 7 and 5 top show traces of butchering, skinning and deliberate fracturing of long bones [26].

This faunal association with cervids and, in particular, deer, elk, roe deer and wild boar is indicative of forest vegetation and marsh environment somewhere in the Pradis Plateau. The presence of bovids and caprids suggest the existence of patchy woodland compatible with the mountain context. Cave bears were well adapted to this kind of environment, and used the cavities for hibernation, as suggested from the faunal assemblage recovered during the last field-campaigns.

## Materials and Methods

### Ethics Statement

All necessary permits were obtained from the Archaeological Superintendence of the Friuli-Venezia Giulia for the described study, which complied with all relevant regulations. The identification numbers of the specimens analyzed are: GRS13SP57**-**89, GRS13SP57**-**138, GRS13SP57**-**153, GRS13SP57**-**125, GRS13SP57**-**37, GRS13SP57**-**11, GRS13SP57-18, GRS13SP57-46, GRS13SP57-2, GRS13SP57**-**4.

Repository information: the specimen is temporary housed at the University of Ferrara, in the Section of Prehistory and Anthropology, Corso Ercole I d’Este Ferrara, Italy, with the permission of the Archaeological Superintendence of the Friuli-Venezia Giulia.

### Samples Selection and Radiocarbon Pretreatment

We selected 10 well preserved thick cortical bone fragments with and without cut marks from each layer. Four bones from layer 7 (three of them with cut marks), four bones from layer 5 (three with cut marks) and two charcoal samples from the hearth SI of layer 6.

Bone collagen was extracted at the Department of Human Evolution, Max Planck Institute for Evolutionary Anthropology (MPI-EVA), Leipzig, Germany, using the ultrafiltration method described in Talamo and Richards [27]. The outer surface of the bone samples was first cleaned by a shot blaster and then 500 mg of bone was taken. The samples were then decalcified in 0.5 M HCl at room temperature until no CO_2_ effervescence was observed, usually for about 4 hours. 0.1 M NaOH was added for 30 minutes to remove humics. The NaOH step was followed by a final 0.5 M HCl step for 15 minutes. The resulting solid was gelatinized following Longin (1971) at pH 3 in a heater block at 75°C for 20 h. The gelatin was then filtered in an Eeze-Filter™ (Elkay Laboratory Products (UK) Ltd.) to remove small (<80 µm) particles. The gelatin was then ultrafiltered with Sartorius “Vivaspin 15” 30 KDa ultrafilters [28]. Prior to use, the filter was cleaned to remove carbon containing humectants [29]. The samples were lyophilized for 48 hours.

The collagen extract was weighed into pre-cleaned tin capsules for quality control of the material. Stable isotopic analysis was evaluated using a ThermiFinnigan Flash EA coupled to a Delta V isotope ratio mass spectrometer.

The two charcoal samples were sent directly to the Klaus-Tschira-AMS facility of the Curt-Engelhorn Centre in Mannheim, Germany, where they were pretreated with the ABA method [30].

## Results and Discussion

### ^14^C Results

At Rio Secco Cave the C:N ratio of all the samples are 3.2 which is fully within the acceptable range (between 2.9 and 3.6), and all of them displayed a high collagen yield, mostly ranging between 2.4 to 8.2%, substantially higher than 1% of weight for the standard limit [31], [32] (Table 1).

**Table 1:** Radiocarbon dates of Rio Secco Cave.

MPI Lab nr.	Square	U.S.	Material	%Coll	δ^13^C	δ^15^N	%C	%N	C:N	AMS Lab nr.	^14^C Age	1σ Err
S-EVA 26233	I 13	6_SI 2	Charcoal	ABA pretreatment						MAMS-15906	28,995	135
S-EVA 26234	I 13e	6_SI 4	Charcoal	ABA pretreatment						MAMS-15907	29,390	135
S-EVA 25353	I14 b	5 top	Bone with cut marks	5.7	−19.6	2.6	43.7	16.1	3.2	MAMS-15230	44,100	660
S-EVA 25355	G14III	5 top I	Bone with cut marks	3.5	−19.5	4.1	40.1	14.7	3.2	MAMS-15231	45,695	790
S-EVA 25356	H14IV	5 top II	Bone	2.4	−20.6	0.8	37.6	13.8	3.2	MAMS-15232	43,210	600
S-EVA 25357	I14II	5 top I	Bone with cut marks	7.6	−22.3	2.1	42.4	15.5	3.2	MAMS-15233	45,740	800
S-EVA 25359	H14h	7	Bone	5.2	−22.2	1.0	42.4	15.6	3.2	MAMS-15235	46,320	1430
S-EVA 25361	H13IV	7	Bone with cut marks	8.2	−21.8	2.4	44.3	16.2	3.2	MAMS-15236	>49,000	
S-EVA 25362	H13IV	7	Bone with cut marks	4.1	−21.8	1.2	41.6	15.3	3.2	MAMS-15237	44,560	1150
S-EVA 25363	H14g	7	Bone with cut marks	5.5	−21.6	1.6	43.9	15.9	3.2	MAMS-15238	44,770	1180

Isotopic values, C:N ratios, amount of collagen extracted (%Coll) refer to the >30 kDa fraction. The results of AMS radiocarbon dating of 10 samples from Rio Secco Cave of layer (U.S.) 6 (Gravettian cultural sequence), layers (U.S.) 5 top and 7 (Mousterian cultural sequence). δ^13^C values are reported relative to the vPDB standard and δ^15^N values are reported relative to the AIR standard.

Once these criteria were evaluated, between 3 and 5 mg of the collagen samples were sent to the Mannheim AMS laboratory (Lab code: MAMS), where they were graphitized and dated [30].

The radiocarbon results are listed in table 1. All dates were corrected for a residual preparation background (generally<0,0025 F ^14^C, equivalent to ca. >48,000 ^14^C years BP) estimated from pretreated ^14^C free bone samples, kindly provided by the ORAU and pretreated in the same way as the archaeological samples.

The uncalibrated radiocarbon dates of late Mousterian (layer 7) range from >49,000 to 44,560 ^14^C years BP. The four dates of layer 5 range from 45,740 to 43,210 ^14^C years BP. The uppermost layer (layer 6), which was identified as a Gravettian layer, ranges from 29,390 to 28,995 ^14^C years BP. There is no discrepancy between the results obtained on bones with cut marks and without cut marks.

### Comparison with Previous ^14^C AMS Results

A series of radiocarbon dates were previously obtained from layers 8, 5 and 6 [24], [25] (Table 2). The two dates in Layer 8 show a strong discrepancy in the results, in fact the ultrafiltered bone gives an age older than 48,000 ^14^C BP but the charcoal result, pretreated with ABOX-SC, displayed an age of 42,000±900 ^14^C BP. The main argument for this difference has to be found, as described above, in the stratigraphic entities of the layer, in fact it contains frequent tiny charcoals of undetermined conifer, small bones and burnt bones. Moreover deformations, removal and various crossings by marmots and other minor bioturbations affect this layer. In addition, a test-pit opened during the last field campaign (summer 2013) had detected no archaeological traces at 1,5 meters underneath this layer, thus excluding possible pollution from older deposits. For this reason we considered the youngest date (OxA-25359 ^14^C Age 42,000±900) as an outlier.

**Table 2:** Previous radiometric dates of Rio Secco Cave obtained in 2002.

Context	Nature	Lab. Ref.	^14^C age BP ±1σErr	Cal. BP 1σ
6, sq.J11, n.3	Charcoal	Poz-41207	27,080±230	31,240–30950
6, sq.J11, n.4	Charcoal	Poz-41208	28,300±260	32,600–31740
5, GRSI	Bone	LTL429A	37,790±360	42,360–41850
8, sq.H11IV, n.17	Charcoal	OxA-25359	42,000±900	46,220–44560
8, sq.H12IV, n.12	Bone	OxA-25336	>48,000	Infinite

Calibrated ages at 1σ error, using OxCal 4.2 [33] and IntCal13 [34].

Layer 5 has produced an age that is too young compared with our new results (LTL-429A, ^14^C Age 37,790±360) [24]. The sample was selected from the test pit investigated in 2002 and at that time it was not possible to recognize bioturbation produced by marmots. This result is not included in the Bayesian model, discussed below.

Two other charcoal samples in layer 6 were dated at Poznan AMS laboratory pretreated using the ABOX-SC method; these results are consistent with our new results. We incorporate them in the Bayesian model for the distribution of ages.

### Discussion of Chronology

**Figure 6:**
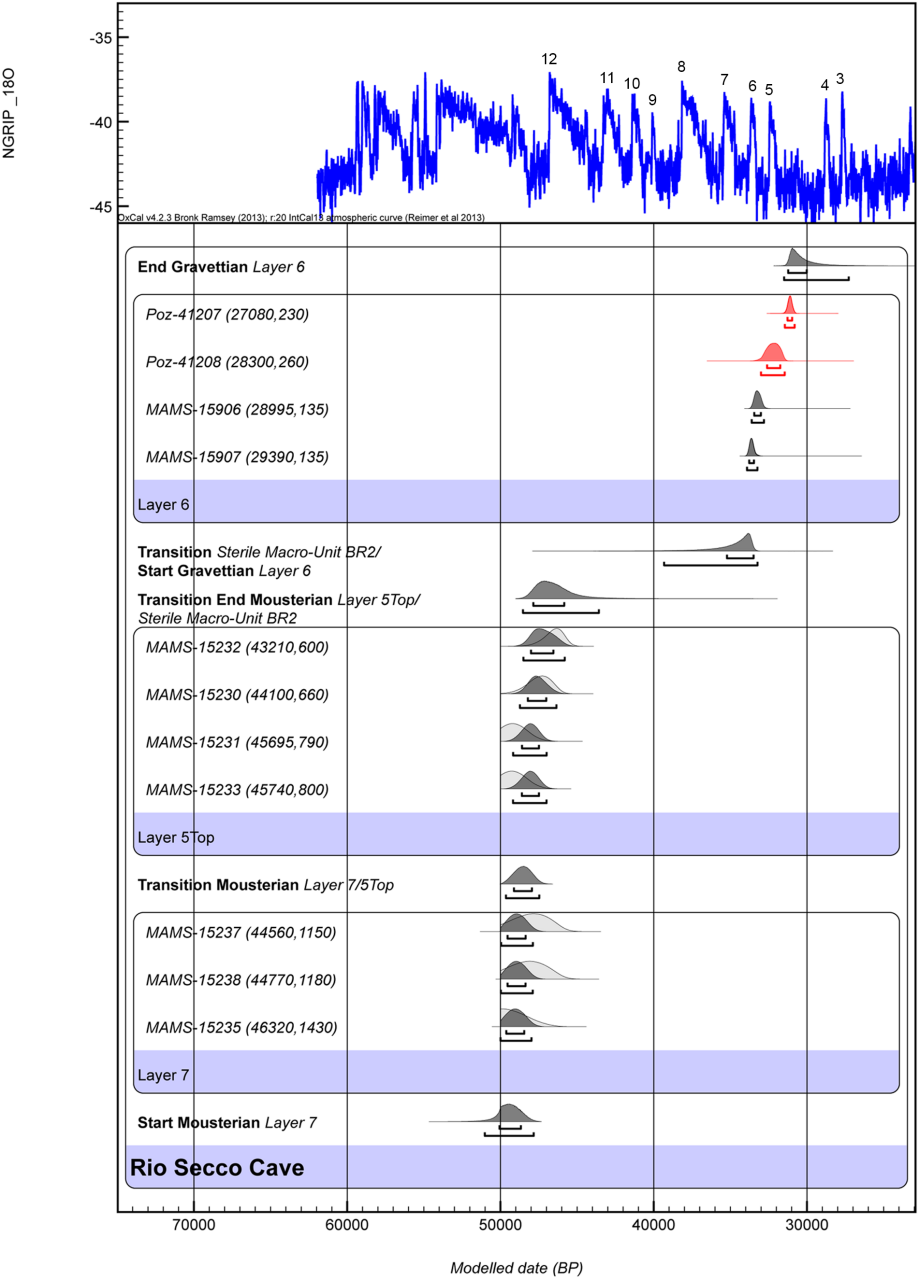
Calibrated ages and boundaries. Calibrated ages and boundaries calculated using OxCal 4.2 [33] and IntCal13 [34]. Rio Secco ages are in black and the previous radiometric results from Poznan (Lab. code Poz-) are in red. The results are linked with the (NGRIP) δ18O climate record.

The radiocarbon dates we produced were calibrated using OxCal 4.2 [33] and IntCal13 [34], (Table 3). The Bayesian model, which was built using the stratigraphic information, includes a sequence of 3 sequential phases, the two Mousterian Layers 7 and 5 top and the Gravettian layer 6 (Figure 6).

**Table 3:** Calibrate boundaries of Rio Secco Cave.

Rio Secco	Modelled Cal BP			
	from	to	from	to
**Indice**	68.20%		95.40%	
A_model 71.3				
**A_overall** 71.9				
**End Gravettian** Layer 6	31,230	30,020	31,500	27,270
**Transition** Sterile Macro-Unit BR2/**Start Gravettian** Layer 6	35,230	33,480	39,330	33,220
**Transition End Mousterian** Layer 5 top/Sterile Macro-Unit BR2	47,860	45,840	48,540	43,570
**Transition Mousterian** Layer 7/5 top	49,120	47,940	49,650	47,460
**Start Mousterian** Layer 7	50,070	48,670	51,020	47,830

Calibrate boundaries provided by OxCal 4.2 [33] using the international calibration curve IntCal 13 [34].

The agreement indices were applied to show how the unmodelled calibrated distribution agrees with the distribution after Bayesian modelling. The agreement index is expected to be above 60% when the dates concord with the stratigraphy. The t-type outlier analysis, performed to detect problematic samples, with prior probabilities set at 0.05, was also incorporated within the Bayesian model [35].

A start calibrated boundary for the lower part of the sequence (Layer 7) at Rio Secco Cave cannot be defined. What we can determine is that the lower level of Layer 7 is older than 49,000 ^14^C BP. With this new determination we can confirm the former date obtained on ultrafiltered bone at ORAU (OxA-25336, ^14^C Age >48,000) in the layer below layer 7 (layer 8) [25] and the only other date (OxA-25359 ^14^C Age 42,000±900) in layer 8 is confirmed to be an outlier. OxCal finds a good agreement index (A_overall = 71.9%), between the full set of finite radiocarbon dates and the stratigraphic information; the results of the outlier detection method confirm ideal posterior probability for all the samples.

The upper boundary of layer 7, calculated by OxCal, ranges from 49,120 to 47,940 cal BP (68.2%); the layer 5 top ranges from 47,940 to 45,840 cal BP (68.2%) (Table 3).

uAround the Alpine regions Neanderthal sites with comparable age ranges are rare [3], [36]. In northern Italy four sites can be considered; Fumane Cave, San Bernardino Cave unit II, Broion Cave and Generosa Cave [3]. In Slovenia on the Šebreljska Plateau the Divje Babe I Cave is contemplated [37] and moving east, in the Drava basin, a layer chronologically consistent with Rio Secco, is found at Vindija Cave [38], [39].

The charcoal samples, from the archaeological horizon US 6_SI located between layers BR2 and BR1 range from 33,480 to 30,020 cal BP (68.2%) (Table 3). These ranges clearly place the upper part of Rio Secco in the early Gravettian period and confirm its archaeological assessment.

It should be noted that the charcoal samples dated at Mannheim yielded consistent age with the previous radiometric dates obtained at Poznan for the same horizon.

Here it is useful to remember that strong progress has been achieved in the last decade on the radiocarbon method. Calibration is now possible back to 50,000 cal BP [34], [40] and claims of fundamental limitations are not justified [41]. Moreover, samples selection and specific pretreatment procedures to remove modern contaminations have been significantly improved [27], [42]–[44].

An accurate sample selection, more specialized pretreatment protocols, the control of isotopic values of bone collagen, in case the samples pretreated were bones and the requirement of several dated samples per layer are fundamental criteria that should be considered in order to establish the radiocarbon chronology of the archaeological sites.

Normally the risk of underestimating the true age of the samples is higher when the samples are at the limit of the radiocarbon method. However the chronological reassessment of Geiβenklösterle, Abri Pataud, Fumane Cave and Mochi rockshelter sites [45]–[48] demonstrated that this problem might occur also between 30,000–20,000 ^14^C years BP.

Bearing in mind this fundamental issue, Rio Secco Cave layer 6 shows the newest radiometric assessment of the Italian late Mid-Upper Palaeolithic. Moreover the comparison with the single dates of layer 23 in Paglicci Cave, permits to ascribe Rio Secco as the oldest Early Gravettian site in Italy.

At this stage of our investigation, the backed pieces and the burins introduced and reduced on site are an expression of short term occupations by hunter gatherers equipped with previously retouched tools made of high quality flints collected outside the Carnic Pre-Alps [25]. However, further investigation is required.

The appearance of the early Gravettian in Europe predates the last phases of the Aurignacian [49]. Although some similarities have been detected with the Ahmarian assemblages of the Near East [49], a local development of the Gravettian technological innovations from the Aurignacian substrate was suggested at Geiβenklösterle in layer AH II [50] and at Abri Pataud in layer 6 [51]. Generally speaking the Gravettian might be interpreted as a macro techno-complex characterized by different and synchronic geographic variants [52]. To the north of the Alps, the key Swabian Gravettian facies include the lithic assemblages of the sites Geiβenklösterle layer AHI, Hohle Fels layer II, Sirgenstein layer II, Brillenhöhle, Weinberghöhlen and Willendorf II layer 5, which are comparable with the Rio Secco age range, (Table S1) [53].

In central Europe between northern Austria, Moravia and southern Poland one finds a second early Gravettian techno-complex, named the Pavlovian, [54], [55]. It is represented at the key sites of Dolní Ve˘stonice II, Pavlov I, Předmostí I and Krems [49], [56], which are contemporaneous with Rio Secco layer 6 (Table S1) [57]. This cultural entity differs from the Swabian Gravettian due to the presence in the toolkit of geometric microliths, micro-burins and Pavlovian points [53].

Furthermore, in the Italian Peninsula local developments of the Gravettian have not been recorded so far [11] and the similarities documented in the lithic assemblages of level 23 of Paglicci Cave and Kostienki 8/II [16], [58] draw attention to the broader Gravettian diffusion from central Europe.

Current evidences make us inclined on the cultural diffusion hypothesis, and the Rio Secco site provides new insight on the two natural corridors used to reach the Italian Peninsula, the Adriatic southern coast from Croatia [9], [20] and the bridge to the north-east from the Carpathian regions. Further researches on the raw materials provenance will shade light on the exploitation of southern or eastern Alpine outcrops determining the foraging radius of these earliest Gravettian groups.

## Conclusion

At the junction between the North Adriatic Plain and the eastern Alps, the chronometric refinement of a new site, Rio Secco Cave, contributes to enhance the investigation of the prehistoric human occupation during the mid-Late Pleistocene. Although not completely explored, Rio Secco Cave fills an important chronological gap and preserves an archive of potential interest for understanding the study of the late Neanderthals, the dispersal of Mid-Upper Palaeolithic populations and the diffusion of the Gravettian culture. Nevertheless, the new set of dates does not cover the millennia of the Middle-Upper Palaeolithic transition in the second half of MIS3, a period chronometrically secured from key-sequences in neighboring regions [45]. Before claiming human ecological or economic factors leading to this dearth of evidence, more data are required from the study of the stratigraphic sequence. The detection of possible stops in the gradation processes of the cave deposit, which may have produced alterations, consolidations, weathering or, alternatively erosions, could explain the complete removal of traces of Aurignacian occupations.

The continued implementation of the project with fieldwork and laboratory studies will provide new elements necessary to better understand the settlements in this area, previously considered so marginal in comparison with the North Adriatic Plain, extending towards the south. At the present stage of research, the Gravettian archaeological record at Rio Secco Cave is scarce compared with the Mousterian one, due to the thinning of layer 6 and its partial reworking produced by illegal excavations in the inner cavity. We cannot exclude that the rockfall that occurred after the late Middle Palaeolithic induced the Gravettian foragers to place their settlement under the present-day rockshelter just in front of the cave entrance. Nevertheless, the few flint artifacts give economic hints of potential interest. The ^14^C results show that the excavated archaeological horizon Layer 6 belongs to the early Gravettian time period. At the present state of knowledge, with our new ^14^C dates, the emergence of the early Gravettian in eastern Italy is contemporaneous with the Swabian Gravettian and the Pavlovian.

The broad expansion of Swabian Gravettian and Pavlovian techno-complexes is explained by high mobility patterns of the hunter-gatherers with transport of exogenous raw materials up to 200 km [50], [59]–[61]. In this scenario the mechanism of the culturally mediated migration might have facilitated the diffusion of the Gravettian innovations and their assimilations in the technical behaviors in the neighboring regions.

Although the absence of diagnostic lithic tools at Rio Secco Cave layer 6 doesn’t allow a correlation of the lithic assemblages with the central European techno-complexes, the radiometric dates support the hypothesis of dispersal of the Swabian Gravettian probably from the eastern Austria (Figure 1). In the neighborhood of Rio Secco Cave there are several gorges originating from a combination of tectonic uplift, karstic processes and run-off erosion. Along these gorges, several shelters and caves were formed in the walls and many others at the base of rock walls. Only a few of them (Verdi Caves and Clusantin Cave) have been explored for the presence of Pleistocene fills and have yielded Mousterian and late Epigravettian evidence for human frequentation [62], [63]. This situation suggests that the absence of the Mid-Upper Palaeolithic in the eastern Alps may reflect a lack of the archaeological investigation rather than a gap in prehistoric human presence. So far Rio Secco Cave yields new insights for the presence of the last Neanderthals and the spread of Gravettian populations into the junction between the plain and the alpine regions.

## Supporting Information

Table S1 Radiocarbon dates on key Gravettian sites [1]–[12].
(DOCX)
