# Supplementary material for: Detecting Human Presence at the Border of the Northeastern Italian Pre-Alps. 14C Dating at Rio Secco Cave as Expression of the First Gravettian and the Late Mousterian in the Northern Adriatic Region
Source: PLoS One. 2014 Apr 23;9(4):e95376. doi: 10.1371/journal.pone.0095376 (PMC3997387; doi:10.1371/journal.pone.0095376)
Supplement: Table S1 — Radiocarbon dates on key Gravettian sites [1]–[12]. (DOCX) [file pone.0095376.s001.docx]

| **Country** | **Site** | **Layer** | **Lab Nr.** | **^14^C Age** | **1σ Err** | **References** |
| --- | --- | --- | --- | --- | --- | --- |
| **Germany** | Geißenklösterle | I | OxA 21740 | 26,420 | 230 | Higham et al. 2012 |
|  | Geißenklösterle | I | OxA 21660 | 27,960 | 290 | Higham et al. 2012 |
|  | Geißenklösterle | I | OxA 21739 | 28,600 | 290 | Higham et al. 2012 |
|  | Geißenklösterle | I | OxA 21661 | 32,900 | 450 | Higham et al. 2012 |
|  | Sirgenstein | II | KIA 13079 | 27,250 | 180 | Conard & Moreau 2004 |
|  | Sirgenstein | III | KIA 13080 | 30,210 | 220 | Conard & Moreau 2004 |
|  | HohleFels | II | OxA 4598 | 26,000 | 360 | Conard & Moreau 2004 |
|  | HohleFels | II | KIA 3503 | 27,030 | 250 | Conard & Moreau 2004 |
|  | HohleFels | II | KIA 17742 | 27,690 | 140 | Conard & Moreau 2004 |
|  | HohleFels | II | KIA 17744 | 27,780 | 150 | Conard & Moreau 2004 |
|  | HohleFels | II | KIA 17743 | 27,830 | 150 | Conard & Moreau 2004 |
|  | HohleFels | II | H856 | 27,840 | 190 | Conard & Moreau 2004 |
|  | HohleFels | II | KIA 17741 | 27,970 | 140 | Conard & Moreau 2004 |
|  | HohleFels | II | H145 | 28,060 | 170 | Conard & Moreau 2004 |
|  | HohleFels | II | H911 | 28,170 | 220 | Conard & Moreau 2004 |
|  | HohleFels | II | H141 | 28,170 | 180 | Conard & Moreau 2004 |
|  | HohleFels | II | OxA 4599 | 28,920 | 440 | Conard & Moreau 2004 |
|  | HohleFels | II | OxA 5007 | 29,550 | 650 | Conard & Moreau 2004 |
|  | HohleFels | II | KIA 8964 | 29,560 | 240 | Conard & Moreau 2004 |
|  | HohleFels | II | KIA 8965 | 30,010 | 220 | Conard & Moreau 2004 |
|  | HohleFels | II | KIA 16040 | 30,640 | 190 | Conard & Moreau 2004 |
|  | Brillenhöhle | VII | B 492 | >25,000 |  | Conard & Moreau 2004 |
|  | Brillenhöhle | VII | KIA 19553 | 25,870 | 230 | Conard & Moreau 2004 |
|  | Brillenhöhle | VII | KIA 19549 | 27,030 | 180 | Conard & Moreau 2004 |
|  | Weinberghöhlen | C | GrN-6059 | 28,265 | 325 | Moreau et al. 2013 |
|  | Weinberghöhlen | C | GrN-5000 | 29,410 | 470 | Moreau et al. 2013 |
| **Czech Republic** | Dolni Vestonice II | 5 | OxA-18038 | 26,460 | 140 | Beresford-Jones et al.2010 and 2011 |
|  | Dolni Vestonice II | 5 | OxA-17811 | 26,770 | 140 | Beresford-Jones et al.2010 and 2011 |
|  | Dolni Vestonice II | 5 | OxA-17814 | 26,850 | 140 | Beresford-Jones et al.2010 and 2011 |
|  | Dolni Vestonice II | 5 | OxA-17813 | 27,080 | 140 | Beresford-Jones et al.2010 and 2011 |
|  | Dolni Vestonice II | 5 | OxA-17810 | 28,050 | 150 | Beresford-Jones et al.2010 and 2011 |
|  | Dolni Vestonice II | 5 | OxA-17809 | 28,310 | 150 | Beresford-Jones et al.2010 and 2011 |
|  | Dolni Vestonice II | 5 | OxA-17853 | 28,450 | 170 | Beresford-Jones et al.2010 and 2011 |
|  | Dolni Vestonice II | 5 | OxA-17812 | 28,550 | 150 | Beresford-Jones et al.2010 and 2011 |
|  | Předmostí I | Cultural Layer | GrN-6801 | 26,870 | 250 | Svodoba et al. 1994 |
|  | Předmostí I | Cultural Layer | GrN-6852 | 26,320 | 240 | Svodoba et al. 1994 |
|  | Pavlov I | Cultural Layer | GrN-1325 | 25,020 | 150 | Damblon et al. 1996 |
|  | Pavlov I | Cultural Layer | GrN-22304 | 25,160 | 170 | Van der Plitch 1997 |
|  | Pavlov I | Cultural Layer | GrA-192 | 25,530 | 110 | Damblon et al. 1996 |
|  | Pavlov I | Cultural Layer | GrN-22305 | 25,840 | 290 | Van der Plitch 1997 |
|  | Pavlov I | Cultural Layer | GIN-104 | 26,000 | 350 | Damblon et al. 1996 |
|  | Pavlov I | Cultural Layer | GrN-20391 | 26,170 | 450 | Damblon et al. 1996 |
|  | Pavlov I | Cultural Layer | GrN-22303 | 26,400 | 310 | Van der Plitch 1997 |
|  | Pavlov I | Cultural Layer | KN-1286 | 26,600 | 500 | Damblon et al. 1996 |
|  | Pavlov I | Cultural Layer | GrN-1272 | 26,620 | 230 | Damblon et al. 1996 |
|  | Pavlov I | Cultural Layer | GrN-19539 | 26,650 | 230 | Damblon et al. 1996 |
|  | Pavlov I | Cultural Layer | GrN-4812 | 26,730 | 250 | Damblon et al. 1996 |
| **Austria** | Krems-Wachtberg | AH5 | VERA-3940 | 28,470 | 280 | Einwögerer et al. 2009 |
|  | Krems-Wachtberg | AH5 | VERA-4535 | 28,700 | 290 | Einwögerer et al. 2009 |
|  | Krems-Wachtberg | AH5 | VERA-3939 | 28,750 | 270 | Einwögerer et al. 2009 |
|  | Willendorf II | 6 | GrA-1016 | 26,150 | 110 | Nigst et al. 2008 |
|  | Willendorf II | 6 | GrA-20768 | 26,500 | 480 | Nigst et al. 2008 |
|  | Willendorf II | 6 | GrN-17803 | 27,600 | 480 | Nigst et al. 2008 |
|  | Willendorf II | 6 | GrA-895 | 27,620 | 230 | Nigst et al. 2008 |
|  | Willendorf II | 6 | GrN-17804 | 28,560 | 520 | Nigst et al. 2008 |
|  | Willendorf II | 5 | GrA-218 | 27,270 | 290 | Nigst et al. 2008 |
|  | Willendorf II | 5 | GrN-11193 | 30,500 | 900 | Nigst et al. 2008 |
|  | Willendorf II | 5 | H-246-231 | 32,000 | 3000 | Nigst et al. 2008 |
|  | Gobelsburg | Foyer | VERA-1768 | 29,600 | 280 | Moreau et al. 2013 |
|  | Gobelsburg | Foyer | VERA-1762 | 29,930 | 280 | Moreau et al. 2013 |
| **Italy** | Paglicci | 18 | AMS? | 20,200 | 305 | Boscato 2007 |
|  | Paglicci | 18 | AMS? | 20,160 | 160 | Boscato 2007 |
|  | Paglicci | 19 | AMS? | 20,730 | 290 | Boscato 2007 |
|  | Paglicci | 20 | AMS? | 21,260 | 340 | Boscato 2007 |
|  | Paglicci | 20 | AMS? | 22,220 | 360 | Boscato 2007 |
|  | Paglicci | 20 | AMS? | 22,630 | 221 | Boscato 2007 |
|  | Paglicci | 21 | AMS? | 23,040 | 380 | Boscato 2007 |
|  | Paglicci | 21 | AMS? | 23,470 | 370 | Boscato 2007 |
|  | Paglicci | 21 | AMS? | 23,750 | 390 | Boscato 2007 |
|  | Paglicci | 21 | AMS? | 24,210 | 410 | Boscato 2007 |
|  | Paglicci | 21 | AMS? | 24,720 | 420 | Boscato 2007 |
|  | Paglicci | 22 | AMS? | 26,800 | 300 | Boscato 2007 |
|  | Paglicci | 23 | AMS? | 28,100 | 400 | Boscato 2007 |
|  | Cala | 1 | AMS? | 26,380 | 260 | Boscato 2007 |
|  | Cala | 3 | AMS? | 26,800 | 320 | Boscato 2007 |
|  | Mochi | D | OxA-19800 | 24,600 | 100 | Douka et al. 2012 |
|  | Rio Secco | 6 | Poz-41207 | 27,080 | 230 | Peresani et al. 2013 and this paper |
|  | Rio Secco | 6 | Poz-41208 | 28,300 | 260 | Peresani et al. 2013 and this paper |
|  | Rio Secco | 6 | MAMS-15906 | 28,995 | 135 | This paper |
|  | Rio Secco | 6 | MAMS-15907 | 29,390 | 135 | This paper |

***Table S1.*** *Radiocarbon dates on key Gravettian sites [1-12]*

**References**

1. Boscato P (2007) Faunes gravettiennes à grands mammifères de l’Italie du Sud : Grotta della Cala (Salerno) et Grotta Paglicci (Foggia). Paléo 19: 109-114.

2. Douka K, Grimaldi S, Boschian G, del Lucchese A, Higham TFG (2012) A new chronostratigraphic framework for the Upper Palaeolithic of Riparo Mochi (Italy). Journal of Human Evolution 62: 286-299.

3. Higham T, Basell L, Jacobi R, Wood R, Bronk Ramsey C, et al. (2012) Τesting models for the beginnings of the Aurignacian and the advent of figurative art and music: The radiocarbon chronology of Geißenklösterle. Journal of Human Evolution 62: 664-676.

4. Einwogerer T, Handel M, Neugebauerer-Maresch C, Simon U, Steier P, et al. (2009) ^14^C Dating of the Upper Paleolithic Site at Krems-Wachtberg, Austria. Radiocarbon 51: 847-865.

5. Beresford-Jones D, Taylor S, Paine C, Pryor A, Svoboda J, et al. (2011) Rapid climate change in the Upper Palaeolithic: the record of charcoal conifer rings from the Gravettian site of Dolní Vĕstonice, Czech Republic. Quaternary Science Reviews 30: 1948-1964.

6. Svoboda J, Ložek V, Svobodová H, Škrdla P (1994) Predmostí after 110 years. Journal of Field Archaeology 21: 457-472.

7. Damblon F, Haesaerts P, Van Der Plicht J (1996) New datings and considerations on the chronology of Upper Palaeolithic sites in the Great Eurasiatic Plain. Préhistoire Européenne (Liège) 9: 177-231.

8. Van Der Plicht J (1997) The Radiocarbon dating. Pavlov I - Northwest, the Upper Paleolithic burial and its settlement context The Dolni Vestonice Studies. Brno: Academy of Sciences of the Czech Republic.

9. Conard NJ, Moreau L (2004) Current Research on the Gravettian of the Swabian Jura. Mitteilungen der Gesellschaft für Urgeschichte 13.

10. Nigst PR, Viola TB, Haesaerts P, Trnka G (2008) Willendorf II. Wiss Mitt Niederösterr Landesmuseum 19: 31-58.

11. Beresford-Jones DG, Johnson K, Pullen AG, Pryor AJE, Svoboda J, et al. (2010) Burning wood or burning bone? A reconsideration of flotation evidence from Upper Palaeolithic (Gravettian) sites in the Moravian Corridor. Journal of Archaeological Science 37: 2799-2811.

12. Moreau L, Jöris O (2013) La fin de l’Aurignacien. Au sujet de la position chronologique de la station de plein air de Breitenbach dans le contexte du Paléolithique supérieur ancien en Europe centrale. Mémoire de la Société préhistorique française LVI.
